# Mitochondrial genomes do not appear to regulate flowering pattern/reproductive strategy in *Cannabis sativa*

**DOI:** 10.1093/aobpla/plab068

**Published:** 2021-10-30

**Authors:** Ziv Attia, Cloe Pogoda, Daniela Vergara, Nolan C Kane

**Affiliations:** Ecology and Evolutionary and Biology, University of Colorado, Boulder, 1900 Pleasant Street, Boulder, CO 80302, USA

**Keywords:** Genome assembly, haplotype network, hemp, marijuana, reproductive strategy

## Abstract

Currently, the amount of genetic data for *Cannabis* is lacking due to the illegal nature of the plant. Our study used 73 *Cannabis sativa* whole-genome shotgun libraries to reveal eight different mtDNA haplotypes. The most common haplotype contained 60 of the 73 samples studied and was composed of only dioecious individuals. However, other haplotypes contained a mix of both mating strategies (i.e. monoecious and dioecious). From these haplotype groupings we further examined the fully annotated mitochondrial genomes of four hemp individuals with different mt haplotypes and recorded gene content, copy number variation and synteny. Our results revealed highly syntenic mitochondrial genomes that contained ~60 identifiable sequences for protein-coding genes, tRNAs and rRNAs and no obvious rearrangements or chimeric genes. We found no clear evidence that modern reproductive patterns are due to simple cytoplasmic male sterility mutations. It is likely the interaction between nuclear genetic components and the X/Y sex chromosomes that determines reproductive strategy. Additionally, we added 50 % more mitochondrial genomes to the publicly available repository.

## Teaser

The mitochondria is an important organ in the cell that has its own DNA, referred to as haplotype. In this study, we compared the mitochondrial haplotypes from 73 different *Cannabis* individuals including marijuana and hemp varieties to understand the diversity of these genomes. We also wanted to establish whether the haplotypes were associated to the mating strategies found in *Cannabis*, dioecious (males and females) or monoecious (hermaphrodites). We found a common haplotype in 82 % of the individuals, but overall, the *Cannabis* mitochondrial genomes were similar with no clear associations between haplotypes and reproductive strategy.

## Introduction

*Cannabis sativa* is an important annual herb which has been cultivated by humans for millennia. It has extensive amounts of phytochemicals that are used in folk medicine (marijuana type *Cannabis*) and also contains cellulosic fibres (hemp type *Cannabis*), which are valuable in the textile industry (Russo 2011; Skoglund *et al.* 2013; Andre *et al.* 2016). Presently in the USA, the plant is being developed for more extensive industrial purposes after the approval of the 2018 Agriculture Improvement Act (Congress 2018). Prejudices surrounding this crop species are lessening and research activity continues to increase. Thus, understanding the genetic nature of the *Cannabis* plant as a modern agricultural crop will help to inform the development of it as a valuable plant.

Even though most cultivated *Cannabis* for medical and recreational purposes is dioecious, i.e. male and female flowers develop on separate plants when grown from seeds, monoecious populations where male and female flowers are present on the same plant also exist, particularly in industrial hemp varieties. Interestingly, it is unknown what form wild *Cannabis* took and whether humans impacted the selection for dioecy or monoecy. Most researchers agree that there may no longer be any wild populations that can be examined (Small and Cronquist 1976; McPartland 2018). However, modern monoecious varieties have been obtained by selection from naturally occurring variants (Bocsa 1958; Westergaard 1958). These monoecious varieties offer several agronomic/industrial advantages when compared to dioecious cultivars, such as higher crop homogeneity and increased seed yield due to self-fertilization during breeding (Faux *et al.* 2016; Salentijn *et al.* 2019). However, monoecy is also associated with some drawbacks, mainly due to inbreeding reducing genetic variation, leading to lower vigour, and slower breeding improvement (Bocsa and Karus 1998). Additionally, when plants produce seeds, they usually devote energy to that process instead of in the production of secondary metabolites such as THCA (delta 9 tetrahydrocannabinolic acid) and CBDA (cannabidiolic acid), making monoecious varieties less desirable for medicinal/recreational uses (Lubell and Brand 2018).

*Cannabis sativa* in the haploid state has nine autosomes and one sex chromosome (either X or Y; Lloyd 1975; Mandolino *et al.* 1999; Barrett 2013; Käfer *et al.* 2017). Females are typically X/X and males are X/Y. Monoecious individuals also exist, and look cytologically like females, X/X (Van Bakel *et al.* 2011; Razumova *et al.* 2016). This is not unusual in plants, where femaleness can be influenced by other mechanisms such as nuclear or cytoplasmic male sterility (CMS), i.e. a male-suppressing genotype. Further mutations can create a plant that has a nuclear genome that appears monoecious, but is phenotypically female, and does not express male characteristics (Charlesworth 2002).

The factors that determine separate sexes in plants have evolved numerous times over evolutionary history, usually from hermaphroditic origins (Charlesworth 2002; Renner *et al.* 2021). Many theoretical models of the transition from hermaphrodites to dioecious individuals suggest that both cytoplasmic and nuclear male sterility can play significant roles (Charlesworth 2002). These mutations allow the production of viable female flowers only (e.g. a dioecious plant) and prevent the natural production of pollen (e.g. a monoecious plant). These mutations arise frequently (Hanson 1991; Balk and Leaver 2001; León *et al.* 2007) and can be strongly favoured in nature and by plant breeders, if the reduction in pollen production is accompanied by an increase in number of seeds (Duvick 1999; Vavdiya *et al.* 2013). The complex nature of plant sex evolution and control is repeated numerous times in the Plantae kingdom. Some interesting examples include papaya (*Carica papaya*), which in its diecious state utilizes the Y chromosome to determine maleness in the XY individual. In contrast, papaya can also be gynodioecious due to a domestication mutation where the Y chromosome becoming a hermaphroditic chromosome (Chae *et al.* 2020). Another example is *Mercurialis annua* which can be both feminized and masculinized depending on which genes are mutated and epigenetic control (Cossard and Pannell 2019; Khadka *et al.* 2019). Two close relatives of *Cannabis*, *Humulus lupulus* and *H. japonicus*, have been extensively studied and it has been shown that sex is controlled by the X to autosome ratio (Divashuk *et al.* 2014). However, given *Cannabis*’ illegal status, research has been inhibited and it is unclear what specifically affects the determination of sexual strategy in *Cannabis*. Understanding the distribution of dioecious and monoecious flowering patterns, within different haplotype groups, therefore, has broad importance to plant biology and agronomy and may shed light on the evolutionary origins and genetic basis of *Cannabis*’ mating strategy.

Here, we compare 73 unique *Cannabis* individuals that represent both monoecious and dioecious reproductive strategies and interrogate their mitochondrial genome content and organization. Mitochondrial genomes are relatively inexpensive to sequence and assemble given their small size and high copy number in the cell, and offer a useful first look at the genetics of an organism. Our goal was to pursue a detailed examination of these mitochondrial genomes, including annotating protein-coding genes, tRNAs and rRNAs, as well as examining copy number variation (CNV), and synteny to identify potential rearrangements. The data produced here are a significant increase (50 %) in the publicly available genetic information for this species that has been previously inaccessible to the research/scientific community.

## Materials and Methods

### Whole-genome shotgun libraries

We used publicly available whole-genome shotgun libraries (bioproject PRJNA310948) sequenced by Illumina™ Nextera (Lynch *et al.* 2016; Vergara *et al.* 2019). These genomes have raw read lengths from 100 to 151 bp. Detailed information regarding DNA extraction, sequencing and library preparation are provided in Lynch *et al.* (2016) and Vergara *et al.* (2019). These libraries included 73 *C. sativa* individuals, with some cultivars represented multiple times (Carmagnola × 6, Chocolope × 2, Durban Poison × 2, Afghan Kush × 6, Feral Nebraska × 2 and Kompolti × 2; **see****Supporting Information—Table S1**). Duplicates were included in our subsequent analyses as a positive control.

### Variant calling

Genomic libraries for 73 *C. sativa* individuals, 67 of them identified in Lynch *et al.* (2016), were processed to remove adapters and low-quality reads by using Trimmomatic v0.39 (Bolger *et al.* 2014) with the following parameters: Illuminaclip: NexteraPE-PE.fa:2:20:10 Leading:20 Trailing:20 Sliding window:4:15 Minlen:100. The resulting FASTQ files were checked for quality using FASTQC (Andrews 2010). The quality-checked, trimmed sequences were then aligned to the *C. sativa* cs10 assembly (GenBank accession GCA_900626175.2) using the Genome Analysis Toolkit (Van der Auwera *et al.* 2013). The resulting variant call file (VCF) table was filtered using vcftools (Danecek *et al.* 2011) to only include single-nucleotide polymorphisms (SNPs) that specifically aligned to the mitochondrion and had quality scores above 100 (--minQ 100; **see****Supporting Information—Table S2**). The cs10 assembly (Grassa *et al.* 2018) was used as it is currently the most complete (as of time of writing), full annotation publicly available for *Cannabis* and allowed for the entire whole-genome libraries to be aligned, which avoided spurious sequence alignment due to similarity between reads for the plastids and nuclear genome.

### Haplotype determination

To determine the major haplotype groups of the 73 *Cannabis* individuals, we used the 1356 SNPs identified between the 73 *C. sativa* individuals that were present in the filtered VCF table. These SNPs were converted into a FASTA consensus sequence using vcf2phylip.py (Ortiz 2019; **see****Supporting Information—Table S3**). The resulting multi-FASTA was analysed using the R package *pegas* (Paradis 2010) in R version 3.5.3 to calculate and plot the unique haplotype groups. Each haplotype group was coloured based upon reproductive type (dioecious, monoecious and unknown). Each of the 73 individuals were assigned to a haplotype group 1–8 **[see****Supporting Information—Table S1****]**.

### Genome assembly

To carefully determine possible differences between the mitochondrial genomes of monoecious and dioecious *Cannabis* individuals, we focused most of our efforts on two representative monoecious and two representative dioecious cultivars. Two of these, Carmagnola (GenBank accession KR059940.1) and Sievers Infinity (GenBank accession KU363807.1), were already assembled/annotated and were obtained from NCBI. The other two, Kompolti (MT361981.1) and Euro Oil (MT557709), were newly assembled and annotated here. In order to assemble Kompolti, and Euro Oil, *de novo* assembly of trimmed reads into scaffolds was performed with SPAdes v3.11.1 (Bankevich *et al.* 2012). Relative position, order and orientation of scaffolds were determined by comparison to available Carmagnola reference genomes. We selected contigs based on read coverage when multiple contigs represented the same genomic region. Contigs were then placed in the correct order and combined by trimming overlapping sequences. Gaps between scaffolds were filled with either raw or trimmed reads that overlapped (e.g. tiling) from the FASTQ files. Once assembled, zPicture was used to validate the assembly and visualize any potential major differences in structure between the reference (Carmagnola) and each assembled mitochondrial genome (Ovcharenko *et al.* 2004). Additionally, samtools *tView* was used to confirm the absence of chimerically assembled genomes, as well as the presence of high-quality SNPs and/or INDELs (insertions and/or deletions) in the genome. If the mapped reads supported these assembly errors, modifications were made to the FASTA files as needed (Li 2011).

### Mitochondrial genome annotation

Annotations of genomic features (protein-coding sequences, tRNAs and rRNAs) were initiated using GeSeq (Tillich *et al.* 2017) to find approximate locations of the predicted gene features. In order to identify all possible tRNAs, we used tRNAscan-SE 2.0. Additionally, genes not automatically identified using GeSeq were found by using nucleotide and translated protein sequences that were extracted from the reference Carmagnola mitochondrial genome from NCBI. We used BLAST (blastn and blastx) to identify regions with homology to these known sequences in our newly assembled FASTA sequences and subsequently annotated any missing features. Annotations were then completed in NCBI’s Sequin 15.50 (Bethesda, MD) and submitted to GenBank for publication.

### Comparative synteny analysis

The comparative positions of genes and reorganization within mitochondrial genomes based on orthologous relationships were plotted using the GUI program MAUVE with default settings (Darling *et al.* 2004). Comparisons were made between two representative dioecious (Carmagnola and Kompolti) and two monoecious (Sievers Infinity and Euro Oil) individuals.

### Copy number variation

To interrogate the CNV between two representative monoecious and dioecious individuals, we divided the calculated coverage by depth at every position in the genome. Specifically, we determined coverage by reporting the number of mapped reads (samtools view -c -f) and the depth at every position in the sorted.bam file (samtools depth). Coverage was estimated as (# mapped reads) * (read length)/(mitochondrial genome length). Read length was 150 bp for all four haplotypes. Copy number variation was then determined from the formula (coverage)/(depth at every position). These values were then normalized by dividing the CNV at each position by the average of the CNV of the entire genome. Next, we used the GenBank annotation files to denote the boundaries of each gene and their associated exons. We report the normalized CNV values for each gene’s exon in **Supporting Information—Table S4**.

## Results

### Variant calling and haplotype network for 73 *Cannabis* individuals

**Figure 1.**
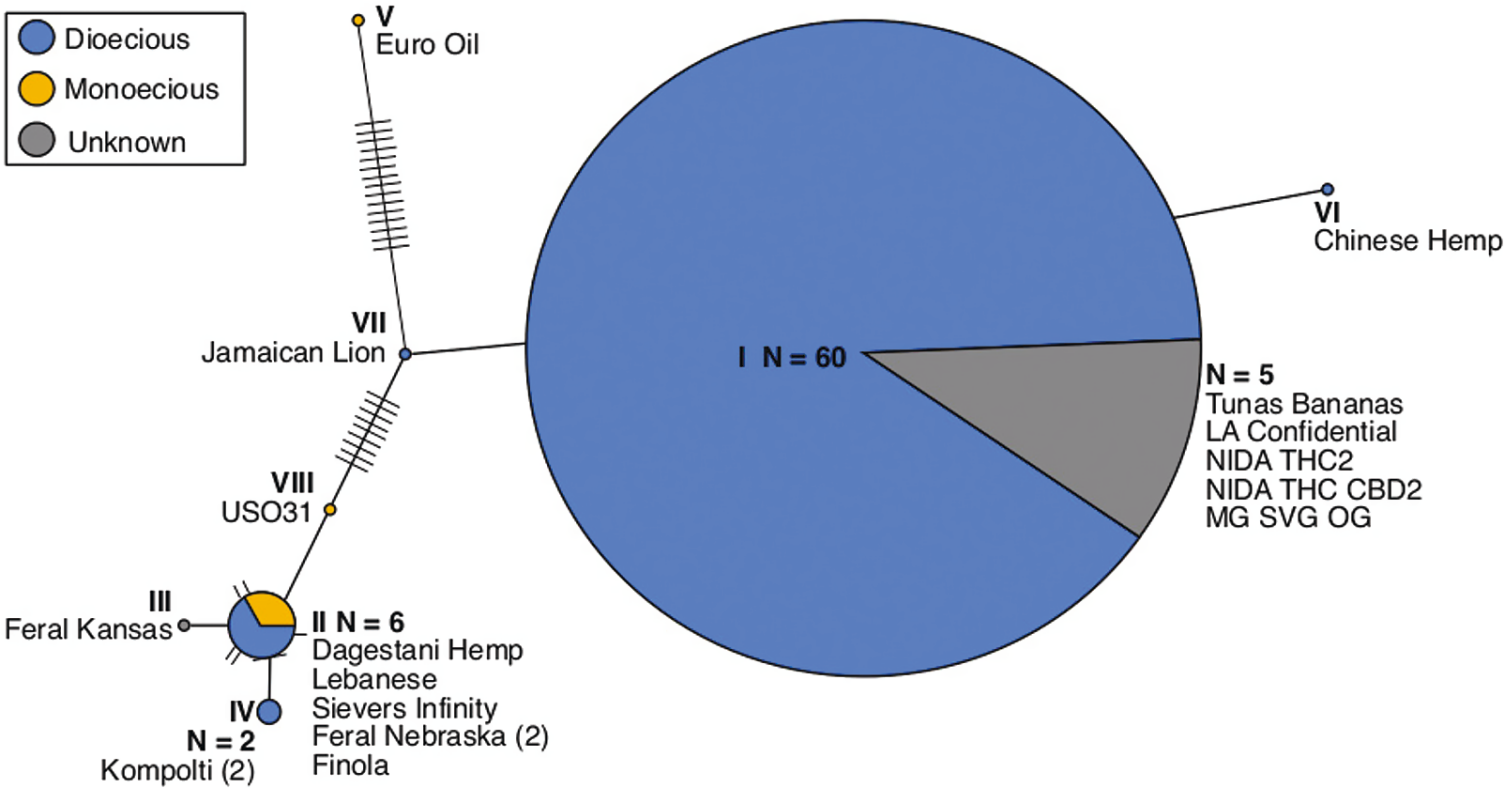
Haplotype network of 73 *Cannabis* individuals (duplicate cultivars are included in the sample size *n*). The total number of individuals (*n*), or the individual’s name is given if it is a group of one. Dioecious sex strategy is represented by blue, monoecious by yellow and unknown is given in grey.

Alignment of the 73 *C. sativa* individuals to the cs10 assembly reference genome (as it is currently the most complete publicly available assembly; Grassa *et al.* 2018) identified a total of 1356 SNPs **[see****Supporting Information—Table S2****]**. Haplotype network prediction using a consensus sequence based on the SNPs **[see****Supporting Information—Table S3****]** produced eight distinct groups. The haplotype groupings are coloured based upon reproductive strategy (Fig. 1; **see****Supporting Information—Table S1**). The largest group contained 60 of the 73 individuals and showed only the dioecious sex strategy (there were also five individuals that the sex strategy is not yet determined). However, this haplotype grouping suggests that those unknown individuals are likely also dioecious. The second largest group contained six individuals of which two (Dagastani hemp and Sievers Infinity) are monoecious and four (Finola, Feral Nebraska × 2 and Lebanese) dioecious. Duplicate cultivars (Carmagnola × 6, Chocolope × 2, Durban Poison × 2, Afghan Kush × 6, Feral Nebraska × 2 and Kompolti × 2) were assigned to the same haplotype groups (group I, I, I, I, II and IV, respectively) as positive controls. Given this haplotype map and our interest in establishing if the mitochondrial genome might affect reproductive strategy, we focused our analysis on four different hemp individuals. We chose two representative monoecious and two representative dioecious genomes for our subsequent analyses **[see****Supporting Information—Fig. S1****]**. The first individual was Carmagnola as it was part of the major haplotype group I and is known to be strongly dioecious (Small 2016). The second dioecious individual selected was Kompolti as it is the most distinct representative of that sex strategy based on our haplotype network. Similarly, we chose Sievers Infinity as a representative monoecious individual, because it is part of the second largest haplotype, group II, and Euro Oil as it is the most distinct monoecious individual based on our haplotype groupings.

### Synteny

**Figure 2.**
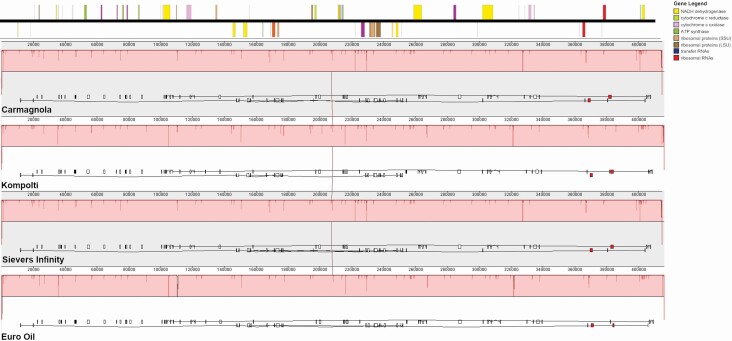
Synteny plot of four hemp haplotypes: Carmagnola (KR_059940), Kompolti (MT361981.1), Euro Oil (MT557709) and Sievers Infinity (KU363807.1). Genetic content for the reference (Carmagnola) is shown at the top of the figure and the figure legend (top right) shows what each coloured object represents. All haplotypes were of similar size (400 kb) and they are aligned to each other.

To begin investigating the possible role of rearrangements of the mitochondrial genome in the potential CMS phenotype, we compared synteny between our four representative individuals. Synteny is shown in Fig. 2 and is well conserved among the four hemp haplotypes. We specifically examined Carmagnola (dioecious) vs. Kompolti (dioecious), Kompolti (dioecious) vs. Euro Oil (Monoecious) and Euro Oil (monoecious) vs. Sievers Infinity (monoecious). All four genomes were aligned to begin with the same nucleotide sequence to ensure appropriate alignment of the genomes. It is clear that there are no rearrangements between any of the individuals and specifically no major or minor difference between the dioecious and monoecious groups was observed **[see****Supporting Information—Fig. S2****]**.

### Mitochondrial genomic content

To shed light on any potential differences in mitochondrial genome content of the *Cannabis* mitochondrial genomes used here, we compared four different hemp mitochondrial genomes: two representative monoecious and two representative dioecious individuals. The mitochondrial gene content was highly similar between the four mitochondrial genomes we analysed. We identified ~60 genes in each mitochondrial genome including protein-coding genes, tRNAs and rRNAs. Forty-two protein-coding genes previously associated with the CMS phenotype were present in all these genomes and are closely compared in Table 1, with only minor observable differences in some cases, such as different numbers of exons or strand orientation of a given gene. These differences, however, could be due to slight variability in annotation rather than underlying genetic differences because genome-wide, nucleotide similarity for the assembled mitochondrial genomes (i.e. FASTA sequences) was 100 % for Carmagnola vs. Sievers Infinity, 99.94 % for Carmagnola vs. Kompolti and 99.96 % for Carmagnola vs. Euro Oil. The differences between dioecious (Carmagnola) and monoecious (Sievers Infinity and Kompolti) and whether they occurred in a coding region or not are presented in Table 2. Out of 25 potential SNPs and INDELs that were different between the Carmagnola representative dioecious individual and both our monoecious individuals (Euro Oil and Sievers Infinity), there were only five that occurred near an annotated gene, with only one (an insertion of AG at position 26 7314 bp) occurring in an exon of nad7 in Carmagnola. However, alignment of this protein did not reveal any amino acid changes, as this insertion occurs outside the coding regions in both the dioecious individuals. GC percentage was identical between the four haplotypes and was found to be 45.6 %.

**Table 1.** Gene presence/absence for the two representative dioecious individuals (Carmagnola and Kompolti) and two representative monoecious individuals (Sievers Infinity and Euro Oil). Number of exons for each gene, tRNA, and rRNA are given as well as if it was on the plus or minus strand. Percentage of coding sequence on the plus strand and GC content is given for each genome.

Gene/haplotype	Carmagnola		Kompolti		Sievers Infinity		Euro Oil	
	Number of exons	Strands	Number of exons	Strands	Number of exons	Strands	Number of exons	Strands
ATP1	1	Plus	1	Plus	1	Plus	1	Plus
ATP4	1	Plus	1	Plus	1	Plus	1	Plus
ATP6	1	Plus	1	Plus	1	Plus	1	Plus
ATP8	1	Plus	1	Plus	1	Plus	1	Plus
ATP9	1	Plus	1	Minus	1	Minus	1	Minus
CcmB	1	Plus	1	Plus	1	Plus	1	Plus
ccmc	1	Plus	1	Plus	1	Plus	1	Plus
ccmfc	2	Minus	2	Minus	2	Minus	1	Minus
ccmfn	1	Plus	1	Plus	1	Plus	1	Plus
cob	1	Plus	1	Plus	1	Plus	1	Plus
cox1	1	Plus	1	Plus	1	Plus	1	Plus
cox2	2	Plus	2	Plus	2	Plus	2	Plus
cox3	1	Plus	1	Plus	1	Plus	1	Plus
matr	1	Plus	1	Minus	1	Minus	1	Minus
mttB	1	Plus	1	Plus	1	Plus	1	Plus
nad1	5	Minus	4	Plus	5	Minus	5	Minus
nad2	5	Plus	5	Plus	4	Plus	5	Plus
nad3	1	Plus	1	Plus	1	Plus	1	Plus
nad4	3	Plus	3	Plus	3	Plus	3	Plus
nad4L	1	Plus	1	Plus	1	Plus	1	Plus
nad5	5	Minus	4	Minus	2	Minus	4	Minus
nad6	1	Plus	1	Minus	1	Minus	1	Minus
nad7	4	Plus	4	Minus	4	Plus	4	Plus
nad9	1	Plus	1	Plus	1	Minus	1	Plus
rpl16	1	Minus	1	Minus	1	Minus	1	Minus
rpl2	2	Minus	2	Minus	2	Minus	2	Minus
rps12	1	Plus	1	Plus	1	Plus	1	Plus
rps13	1	Plus	1	Plus	1	Plus	1	Plus
rps3	2	Minus	2	Minus	2	Minus	2	Minus
rps4	1	Plus	1	Plus	1	Plus	1	Plus
rps7	1	Plus	1	Plus	1	Plus	1	Plus
rrn18	1	Plus	1	Plus	NA	NA	1	Plus
rrn5	1	Minus	1	Plus	1	Minus	1	Plus
trnD	1	Minus	1	Plus	1	Minus	1	Plus
trnE	1	Plus	1	Plus	1	Plus	1	Plus
trnF	1	Plus	1	Plus	1	Plus	1	Plus
trnK	1	Minus	×2	Plus	1	Plus	×2	Plus
trnP	1	Plus	×2	Plus	1	Plus	×2	Plus
trnQ	1	Plus	1	Plus	1	Plus	1	Plus
trnS	1	Plus	×2	Plus	1	Plus	×2	Plus
trnW	1	Plus	1	Plus	1	Plus	1	Plus
trnY	1	Plus	1	Plus	1	Plus	1	Plus
GC content %	45.6		45.6		45.6		45.6	
Genes on plus strand %	78.6		80.0		69.0		80.0	

**Table 2.** Single-nucleotide polymorphism (SNP) and INDEL differences between the reference dioecious individual (Carmagnola) and the alternate monoecious individuals (Euro Oil and Sievers Infinity). Position is given in bp for the reference Carmagnola annotation. The gene feature corresponds to the reference Carmagnola annotation.

Position (bp) in Carmagnola	Reference (dioecious)	Alternate (monoecious)	Gene feature in Carmagnola
3208	C	T	Non-coding
56 571	C	A	Non-coding
74 900	CT	C	Non-coding
122 196	C	A	Non-coding
144 424	A	G	Non-coding
150 582	G	T	Non-coding
167 988	C	A	Intron trans-spliced nad2
213 604	A	T	5′ Leader sequence nad3
247 699	C	G	5′ Leader sequence nad6
256 390	T	G	Non-coding
257 100	C	G	Non-coding
267 314	CAG	CAGAG	Exon nad7
277 630	T	G	Non-coding
295 239	A	T	Non-coding
306 408	T	C	Intron nad4
318 064	C	A	Non-coding
331 459	A	C	Non-coding
363 735	T	G	Non-coding
378 146	A	C	Non-coding
384 437	AGGG	AGGGG	Non-coding
400 445	A	C	Non-coding
401 584	C	G	Non-coding
404 499	G	T	Non-coding
413 301	ATC	ATCGTTC	Non-coding
413 302	TC	TCGTCC	Non-coding

### Copy number variation

**Figure 3.**
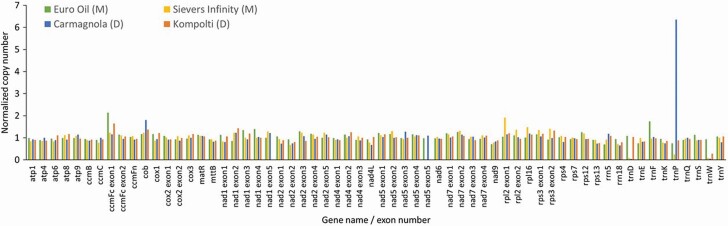
Copy number variation (CNV) for Carmagnola, Sievers Infinity, Kompolti and Euro Oil hemp haplotypes. Values were normalized by dividing by the average CNV of each individual mitochondrial genome.

Copy number variation was similar across the monoecious and dioecious hemp individuals and 42 genes analysed (Fig. 3). The average CNV was ~1. The only outlier was the tRNA trnP (Proline), which has much higher copy number only in the Carmagnola mitochondrial genome.

## Discussion

This study takes a detailed look at different *Cannabis* haplotypes to determine any potential differences in the mitochondrial genomes for these various cultivars. Using 73 *C. sativa* individuals aligned to the cs10 (Grassa *et al.* 2018) reference genome, we identified eight distinct haplotypes, with most of the dioecious individuals (60) falling within haplotype group I (Fig. 1). We chose to closely examine four separate individuals, two (Kompolti and Euro Oil) of which were newly assembled and annotated here. The first of the four we examined, Carmagnola, was part of the main haplotype group I and was dioecious. The second, Sievers Infinity, fell within haplotype group II and was monoecious. All individuals examined are hemp varieties of *C. sativa* and are cultivated for industrial purposes. Carmagnola is strongly dioecious, existing always as separate male and female plants. Kompolti is also dioecious but can express flowers of either sex on the same plant, with the use of chemicals (e.g. Ag^+^ ions and gibberellins; Ram and Jaiswal 1972; Atsmon and Tabbak 1979; Ram and Sett 1982). In contrast, Sievers Infinity and Euro Oil are monoecious **[see****Supporting Information—Table S1****]**. Our analyses revealed that the mitochondrial genomes of all four individuals were remarkably similar even though they belonged to different haplotype groups. The genetic content, SNPs/INDELs, CNV and synteny were all highly conserved. This observation is very close to previous research that looked for differences in the chloroplast genomes of *Cannabis* and found that they too were highly similar and conserved (Vergara *et al.* 2015; Roman *et al.* 2019).

There are two types of sterility possible in plants: mitochondrial- or nuclear-encoded. Mitochondrial-encoded sequences conferring CMS are commonly observed in plant species and are the result of genomic conflict between the mitochondrial and nuclear genomes (Horn *et al.* 2014; Touzet and Meyer 2014). In contrast, nuclear, or genic, male sterility has been associated with 20 nuclear-associated gene mutations (Neuffer *et al.* 1997) and it is also common in flowering plants (Gabay-Laughnan and Laughnan 1994). Our haplotype group results suggest that a single, ancient origin of monoecy from a dioecious ancestor is not supported. If dioecy were ancestral as suggested by previous research (Kovalchuk *et al.* 2020), we would expect more diversity in those dioecious individuals and not the single large haplotype observed. Group I of our haplotype network contains 60 individuals and suggests that instead there was a recent selective sweep specific to this mitochondrial type, but it is not the only cytotype associated with dioecy. The lack of observed diversity in both the mitochondrial and chloroplast genomes is in direct contrast to the nuclear genome diversity in these individuals (Lynch *et al.* 2016). Our genetic data suggest that instead of a simple CMS mutation controlling reproductive strategy there has been a fairly complex evolution of dioecy vs. monoecy in *Cannabis*, perhaps involving several distinct mutations and likely involving the nuclear genome.

## Conclusions

As the nature of *Cannabis* continues to evolve towards being an accepted agricultural crop, more information about agronomic traits is required. This crucial information will provide valuable tools for breeding hemp. Currently, most *Cannabis* for medical and recreational purposes is propagated vegetatively, and stocks of homogenous seed in the USA do not exist. *Cannabis* lacks the genetic and genomic tools available, for most important agricultural crops, due to its illegal status (Vergara *et al.* 2016), and the absence of basic resources such as public isogenic germplasm collections hinders the improvement and development of cultivars. Crop improvement specialists require creative and collaborative solutions to overcome these issues due to years of scientific neglect. Therefore, in order to make *Cannabis* more appealing and widely available to commercial farmers, ‘true breeding’ (e.g. F1 hybrids) approaches are necessary. In addition, focused crossing will allow the development of biparental populations and introgression lines that facilitate sophisticated genomic approaches, such as genome-wide association studies. These methods are well established and used in other agricultural crops like sunflower, corn and tomato (Vear 2016; Bauchet *et al.* 2017; Darrah *et al.* 2019). This type of research has important implications for the medical, recreational and industrial industries that rely on *Cannabis*. Gaining legal acceptance for the scientific study of this plant, given the growing public acceptance, will be a huge step forward in the process of making *Cannabis* into a profitable, accessible and modern crop. These data presented here offer a first step in the genetic interrogation and understanding the enigmatic sexual strategies of this useful and controversial plant.

## Supporting Information

The following additional information is available in the online version of this article—

**Table S1.** Cultivar name, reproductive type (D for dioecious, H for Hermaphrodite (monoecious)) and haplotype group number for the 73 individual *Cannabis sativa* samples used here. Duplicate cultivar names are provided. Haplotype group number is 1–8 and corresponds to Fig. 1 in the main text.

**Table S2.** Variant call format table (SNPs and INDELs) for the 73 *Cannabis sativa* cultivars aligned to the reference cs10 (VCF was created using Genome Analysis Toolkit (GATK)).

**Table S3.** FASTA consensus sequence for each of the 73 *Cannabis sativa* cultivars aligned to the reference cs10 (Grassa *et al.* 2018). A total of 1356 SNPs are included for each individual.

**Table S4.** The normalized copy number variation (CNV) values for each gene’s exon for the four hemp haplotypes.

**Figure S1.** Genomic content for the Carmagnola (KR_059940) and Sievers Infinity (KU363807.1) as well as the two newly sequenced, assembled and annotated *Cannabis sativa* hemp Kompolti (MT361980.1) and Euro Oil (MT557709) mitochondrial genomes.

**Figure S2.** Dot plot synteny comparisons performed via NCBI’s blastn.

plab068_suppl_Supplementary_Figures

plab068_suppl_Supplementary_Tables

## Data Availability

All mitochondrial genomes are publicly available from NCBI: Carmagnola (KR_059940), Sievers Infinity (KU363807.1), Kompolti (MT361980.1) and Euro Oil (MT557709—temporary accession number, awaiting final publication).

## Notes

**Evolution & Diversity. Chief Editor**: Jeremy Beaulieu
